# Differences in organization of care are associated with mortality, severe complication and failure to rescue in emergency colon cancer surgery

**DOI:** 10.1093/intqhc/mzab038

**Published:** 2021-03-02

**Authors:** Daniëlle D Huijts, Jan willem T Dekker, Leti van bodegom-vos, Julia T van groningen, Esther Bastiaannet, Perla J Marang-van de mheen

**Affiliations:** Department of Biomedical Data Sciences, Medical Decision Making, Leiden University Medical Center, Albinusdreef 2, Leiden 2333 ZA, The Netherlands; Department of Surgery, Reinier de Graaf Group, Reinier de Graafweg 5, Delft 2600 GA, The Netherlands; Department of Biomedical Data Sciences, Medical Decision Making, Leiden University Medical Center, Albinusdreef 2, Leiden 2333 ZA, The Netherlands; Dutch Institute for Clinical Auditing, Rijnsburgerweg 10, Leiden 2333 ZA, The Netherlands; Department of Surgery, Leiden University Medical Center, Albinusdreef 2, Leiden 2333 ZA, The Netherlands; Department of Surgery, Leiden University Medical Center, Albinusdreef 2, Leiden 2333 ZA, The Netherlands; Department of Biomedical Data Sciences, Medical Decision Making, Leiden University Medical Center, Albinusdreef 2, Leiden 2333 ZA, The Netherlands

**Keywords:** colon cancer surgery, risk factors, quality of care

## Abstract

**Background:**

Emergency colon cancer surgery is associated with increased mortality and complication risk, which can be due to differences in the organization of hospital care. This study aimed.

**Objective:**

To explore which structural factors in the preoperative, perioperative and postoperative periods influence outcomes after emergency colon cancer surgery.

**Methods:**

An observational study was performed in 30 Dutch hospitals. Medical records from 1738 patients operated in the period 2012 till 2015 were reviewed on the type of referral, intensive care unit (ICU) level, surgeon specialization and experience, duration of surgery and operating room time, blood loss, stay on specialized postoperative ward, complication occurrence, reintervention and day of surgery and linked to case-mix data available in the Dutch Colorectal Audit. Multivariate logistic regression analysis was used to estimate the influence of these factors on 30-day mortality, severe complication and failure to rescue (FTR), after adjustment for case-mix.

**Results:**

Patients operated by a non-Gastro intestinal/oncology specialized surgeon have significantly increased mortality (Odds Ratio (OR) 2.28 [95% confidence interval (95% CI) 1.23–4.23]) and severe complication risk (OR 1.61 [95% CI 1.08–2.39]). Also, duration of stay in the operating room was significantly associated with increased risk on severe complication (OR 1.03 [95% CI 1.01–1.06]). Patients admitted to a non-specialized ward have significantly increased mortality (OR 2.25 [95% CI 1.46–3.47]) and FTR risk (OR 2.39 [95% CI 1.52–3.75]). A low ICU level (basic ICU) was associated with a lower severe complication risk (OR 0.72 [95% CI 0.52–1.00]). Surgery on Tuesday was associated with a higher mortality risk (OR 2.82 [95% CI 1.24–6.40]) and a severe complication risk (OR 1.77, [95% CI 1.19–2.65]).

**Conclusion:**

This study identified a non-specialized surgeon and ward, operating room, time and day of surgery to be risk factors for worse outcomes in emergency colon cancer surgery.

## Introduction

Colon cancer surgery is a commonly performed procedure; nevertheless, among emergency cases, it is associated with a high risk on mortality and complications [1, 2]. Reducing this high risk remains an important objective for quality improvement initiatives. Previous studies identified several patient factors to increase the risk on mortality and complications in colorectal cancer surgery [3–5]. However, other factors including a different organization of care in emergency colorectal cancer surgery may be associated with increased risks and thereby relevant to improve quality of care.

Quality of care, as described by the Donabedian model [6], is a combination of structural factors, process and outcomes. Structural factors, such as the hospital size, level of intensive care unit (ICU) and nurse–patient ratio, have been reported to influence outcomes [7, 8]. However, the study by Sheetz *et al.* [9] showed that some of the included structural factors, together with patient characteristics and surgical volume, could only explain part of the variability in death after a severe complication (failure to rescue (FTR)) following major surgery. They suggested micro-system characteristics rather than the above macro-level characteristics to be important in improving outcomes.

As a proxy for a possible different organization, several recent studies have investigated whether there is inferior quality of care during weekends compared to weekdays and if this explains the weekend effect as found in previous studies [10, 11]. In the Netherlands, a recent study on emergency colorectal cancer surgery, using prospectively collected clinical data from the Dutch ColoRectal Audit (DCRA), showed worse outcomes in mortality and severe complication after weekend emergency surgery for colon cancer [12]. Part of the possible explanation for the worse outcomes observed in surgery during weekends is a difference in organization affecting the quality of care [10, 11] around surgery or during the postoperative recovery period. However, as day of surgery is only a proxy for possible organizational differences, the question remains whether we can more specifically identify structural factors associated with worse outcomes.

The aim of the current study therefore was to investigate which structural factors related to the preoperative, perioperative and postoperative care influence outcomes after emergency colon cancer surgery using data from the DCRA and (electronic) medical records. The DCRA is a nationwide quality improvement audit initiated in 2009 for measuring and improving quality of care for colorectal cancer surgery [13].

## Methods

### Study design and setting

An observational study was performed using record review combined with DCRA data. A more detailed description of the DCRA is published elsewhere [13]. All hospitals in the Netherlands (*n* = 88) performing colorectal cancer surgery were asked to participate in this study. In total, 30 hospitals agreed to participate. These hospitals were situated across the 12 provinces in the Netherlands among which 3 were university hospitals, 17 were teaching hospitals and 10 were general hospitals.

### Study population

All patients from the participating hospitals who underwent an emergent or urgent surgical resection of a colon tumour in the period 2012 until 2015 were included (*n* = 1749). Urgent is defined as non-elective surgery but scheduled at least 12 hours before surgery, and emergent is defined as non-elective surgery that needs to be scheduled within 12 hours. In this paper, urgent and emergent are combined and referred to as emergency surgery. Those with synchronous tumours, ‘wait-and-see’ policy, procedures solely performed through transanal endoscopic microsurgery, and patients without information on date of surgery, mortality or location of the primary tumour were excluded (*n* = 3), consistent with previous studies [12]. An additional eight patients were excluded due to double registration in the DCRA with no possibility to identify the correct patient. The medical records of the remaining 1738 patients were reviewed using a pre-defined structured form in the period April—October 2017. The Medical Ethical Committee of the Leiden University Medical Centre declared that no ethical approval was necessary for this type of study under Dutch law and waived the need for informed written consent from patients (P16.243).

### Data collection

Information regarding case-mix variables for the included patients were extracted from the DCRA database, which have been previously identified as important for patients’ outcomes and hospital comparisons [12, 14, 15]. These involved gender, age, BMI, Charlson comorbidity index, American Society of Anaesthesiologists (ASA) classification score, pathological tumour stage, presence of metastasis (yes/no), preoperative tumour complications (yes/no), additional resection for metastasis (yes/no), additional resection for locally advanced tumour (yes/no), location of the primary tumour, urgency (urgent versus emergent) and year of surgery. Preoperative tumour complications include ileus, perforation, anaemia, abscess and other preoperative tumour complications.

For the medical record data collection, patient identification numbers were provided to the participating hospitals by the trusted third party organization of the DCRA. This way, medical records could be reviewed without including identifying information in the data collection to ensure patients anonymity. Data were collected by one researcher on structural factors regarding preoperative, perioperative and postoperative period for the hospital admissions in which the colon tumour resection took place. Preoperative factors included type of hospital referral, duration of symptoms (days) and preoperative admission (days). Perioperative factors included surgeon specialization and working experience (years). Surgeon specialization was defined as gastrointestinal (GI) and/or oncology, vascular, trauma, lung or no specialization such as surgical resident or fellow, based on information from the hospital and the Association of Surgeons in the Netherlands. Other perioperative factors included amount of blood loss (cc), duration of surgery (first surgical incision until final stitch) and operating room time (patient entering until leaving operating room). Postoperative factors included specialization of the ward, ICU level and day of complication occurrence and performing reintervention. ICU level in the Netherlands distinguishes three levels, based on the available capacity, staffing and management [8] with Level 1 (lowest) having intensivists available only during working hours, providing a minimum of six beds and 2.7 FTE ICU nurses per bed. Level 2 and 3 (highest) have 24 hour availability of intensivists, providing a minimum of 12 beds and more ICU staffing.

### Definitions

Outcome measures were mortality, severe complication and failure to rescue. Mortality was defined as postoperative death in the hospital during the same admission or within 30 days after resection. Severe complication was defined as any postoperative complication leading to a prolonged hospital stay of more than 14 days, a re-intervention or mortality. FTR are those cases with a severe complication who die during the same admission or within 30 days after resection. These definitions are used nationwide in all hospitals and have also been used in previous DCRA studies [8, 12, 14, 16, 17].

### Initial data analysis

Initial data analysis was performed following the recommended three main steps: data cleaning of inconsistencies and resolving if possible, data screening of the data properties and reporting of the findings [18]. The data cleaning involved univariate and multivariate checks which identified 231 data entry errors. This corresponds to 0.2% based on the total variables of interests. Of these, 204 entry errors could be corrected and the remaining 27 entry errors were coded as missing. The handling of the overall missing data is explained below.

### Statistical analysis

First, outcomes and case-mix for patients from participating hospitals (*n* = 1738) were compared to those from non-participating hospitals (*n* = 3306), using X^2^ tests. Next, multiple imputation was performed for the handling of missing data after assessing the missing value patterns. This assessment showed an arbitrary missing value patterns and thus no obstacles for using multiple imputation. The fully conditional specification (MCMC [Markov Chain Monte Carlo]) was used for data imputation, meaning that all independent variables were included and/or used as predictor with the total number of imputations set at 100. This created 100 datasets, or simulations, which were pooled to obtain one average value to be included in the analysis. In addition, predictive mean matching was used to ensure that the imputed value was within the range of the available data. After finalizing the multiple imputations, we compared the imputed data with the non-imputed data for means, standard deviations, minimum and maximum values to assess potential differences.

To answer the research question, univariate logistic regression analysis was first performed to examine which case-mix factors and preoperative, perioperative and postoperative variables regarding the organization of care were associated with the outcomes. All variables with a *P*-value below 0.05 were included in the multivariate analysis to examine independent effects of variables adjusted for case-mix. For the multivariate models, goodness of fit was assessed with C-statistics by calculating the area under the curve. This was done for all 100 datasets after which the mean value was calculated. Univariate and multivariate analyses were also performed for cases with complete (non-missing) information to assess whether our handling of missing data has affected the results. All data handling and analyses were carried out in IBM SPSS statistics version 23.

## Results

### Patient characteristics

A total of 1738 patients were included in the analysis of whom 131 patients (7.5%) died during the hospital admission or within 30 days after resection. In 546 patients (31.4%), a severe complication was observed and 131 of these patients (24%) died after a severe complication (Table 1). When comparing outcomes in patients between participating and non-participating hospitals, we found a significantly higher severe complication rate among patients in participating hospitals, but except for a higher rate of neurological complications (2.8% in participating versus 1.4% in non-participating hospitals, *P* < 0.001)), there were no significant differences in others types such as pulmonary or cardiac complications (data not shown). With respect to case-mix, we found patients in participating hospitals more frequently to have lower ASA score, less advanced tumour stage and differences in types of surgical procedure performed (Table 1).

**Table 1:** Patient characteristics and outcomes for emergency colon cancer patients treated in participating versus non-participating hospitals

		Total (*n* = 5044)		Non-participating hospitals (*n* = 3306)		Participating hospitals (*n* = 1738)		Sign
Outcomes and patient characteristics		*n*	%	*n*	%	*n*	%	*P*
Mortality rate		387	7.7	256	7.7	131	7.5	0.794
Severe complication rate		1467	29.1	921	27.9	546	31.4	**0.008**
Failure to rescue rate		387	26.4	256	27.8	131	24.0	0.110
Case-mix		*n*	%	*n*	%	*n*	%	*P*
Gender	Male	2558	50.7	1656	50.1	902	51.9	
	Female	2485	49.3	1649	49.9	836	48.1	0.226
Age (years)	≤60	900	17.8	588	17.8	312	18.0	
	61–70	1461	29.0	960	29.0	501	28.8	
	71–80	1547	30.7	1030	31.2	517	29.7	
	≥81	1135	22.5	727	22.0	408	23.5	0.596
BMI	<18.5	157	3.5	112	3.7	45	3.0	
	18.5–25	2202	49.0	1467	48.9	735	49.3	
	25–30	1549	34.5	1027	34.2	522	35.0	
	30+	585	13.0	397	13.2	188	12.6	0.581
Charlson comorbidity index	Charlson score 0	2605	51.6	1705	51.6	900	51.8	
	Charlson score 1	1156	22.9	778	23.5	378	21.7	
	Charlson score 2+	1283	25.4	823	24.9	460	26.5	0.259
ASA score	I–II	3156	62.7	2032	61.6	1124	64.7	
	III	1608	31.9	1059	32.1	549	31.6	
	IV–V	273	5.4	209	6.3	64	3.7	**0.001**
Tumour stage	Stage X	24	0.5	18	0.5	6	0.3	
	Stage 0–1	44	0.9	30	0.9	14	0.8	
	Stage 2	212	4.2	130	3.9	82	4.7	
	Stage 3	3088	61.2	1985	60.0	1103	63.5	
	Stage 4	1676	33.2	1143	34.6	533	30.7	**0.038**
Metastasis	No	3801	75.4	2490	75.3	1311	75.4	
	Yes	1243	24.6	816	24.7	427	24.6	0.929
Pre-operative	No	440	8.7	278	8.4	162	9.3	
tumour	Yes	4604	91.3	3028	91.6	1576	90.7	0.275
complications	Perforation	496	11.2	337	11.4	159	10.7	
	Ileus	3508	76.9	2319	77.1	1189	76.7	
	Abscess	167	3.8	110	3.7	57	3.8	
	Anaemia	331	7.4	194	6.6	137	9.1	
	Other	316	7.1	221	7.4	95	6.3	
Additional	No	4824	95.6	3156	95.5	1668	96.0	
resection for	Yes	220	4.4	150	4.5	70	4.0	0.400
metastasis								
Additional	No	4316	85.6	2835	85.8	1481	85.2	
resection for	Yes	728	14.4	471	14.2	257	14.8	0.604
locally advanced tumour								
Location primary	Caecum	866	17.2	543	16.4	323	18.6	
tumour	Appendix	46	0.9	30	0.9	16	0.9	
	Ascending colon	675	13.4	439	13.3	236	13.6	
	Hepatic flexure	311	6.2	194	5.9	117	6.7	
	Transverse colon	466	9.2	303	9.2	163	9.4	
	Splenic flexure	309	6.1	210	6.4	99	5.7	
	Descending colon	477	9.5	319	9.6	158	9.1	
	Sigmoid colon	1894	37.5	1268	38.4	626	36.0	0.385
Type of procedure	Ileocecal resection	122	2.4	73	2.2	49	2.8	
	Right hemicolectomy	2002	39.7	1280	38.7	722	41.5	
	Transverse colectomy	128	2.5	85	2.6	43	2.5	
	Left hemicolectomy	806	16.0	557	16.8	249	14.3	
	Sigmoid colectomy/low anterior resection	1746	34.6	1167	35.3	579	33.3	
	Subtotal colectomy	180	3.6	112	3.4	68	3.9	
	Other	60	1.2	32	1.0	28	1.6	**0.022**
Year of surgery	2012	1361	27.0	894	27.0	467	26.9	
	2013	1290	25.6	838	25.3	452	26.0	
	2014	1246	24.7	810	24.5	436	25.1	
	2015	1147	22.7	764	23.1	383	22.0	0.816
Urgency of surgery	Urgent	2323	46.1	1519	45.9	804	46.3	
	Emergent	2721	53.9	1787	54.1	934	53.7	0.832

Bold value: p ≤ 0.05.

### Independent risk factors

Table 2 shows the structural factors significantly associated with the outcomes in univariate analysis and subsequently included in the multivariate analysis. In multivariate analysis, a higher mortality risk was found for patients not operated by a GI or oncology specialized surgeon (OR 2.28, 95% CI 1.23–4.23) and patients not staying on a specialized ward (OR 2.25, 95% CI 1.46–3.47). Having surgery on Tuesday (OR 2.82, 95% CI 1.24–6.40) significantly elevated the mortality risk, whereas weekend surgery was not independently associated with increased mortality.

**Table 2:** Impact of preoperative, perioperative and postoperative organizational factors on mortality, severe complication and FTR

				Mortality^a^		Severe complication^b^		Failure to rescue^c^	
		*n*	%	OR	95% CI	OR	95% CI	OR	95% CI
Preoperative									
Type referral	Own initiative	138	7.9	0.90	0.30–2.67	0.66	0.39–1.12	1.10	0.33–3.61
	General practitioner	872	50.2	1		1		1	
	Ambulance	95	5.5	1.98	0.89–4.42	0.96	0.54–1.71	2.13	0.85–5.39
	Other	633	36.4	1.14	0.72–1.80	1.00	0.77–1.30	1.09	0.66–1.80
Surgery									
Specialization first surgeon^d^	GI and/or Oncology surgeon	1099	63.2	1		1		–	–
	Other surgeon specialization	124	7.1	**2.28**	**1.23–4.23**	**1.61**	**1.08–2.39**	–	–
	Other	515	29.6	1.42	0.89–2.25	1.02	0.79–1.31	–	–
Operating room time (10 minutes)	Mean, median	157.57	147.00	–	–	**1.03**	**1.01–1.06**	–	–
Blood loss (cc)	Mean, median	220	100	–	–	1.00	1.00–1.00	–	–
Postoperative									
Admitted on a specialized GI and/or oncology ward	No	833	47.9	**2.25**	**1.46–3.47**	–	–	**2.39**	**1.52–3.75**
	Yes	905	52.1	1		–	–	1	
Day of complication	No/unknown complication date	974	56.0	**0.41**	**0.25–0.68**	–	–	–	–
	Weekend	165	9.5	0.98	0.49–1.97	–	–	–	–
	Weekday	599	34.5	1		–	–	–	–
Day of surgical reintervention	No surgical reintervention	1517	87.3	**0.35**	**0.20–0.61**	–	–	–	–
	Weekend	55	3.2	1.49	0.55–4.06	–	–	–	–
	Weekday	166	9.6	1		–	–	–	–
Hospital									
ICU level	1 (low)	326	18.8	–	–	**0.72**	**0.52–1.00**	–	–
	2	666	38.3	–	–	1.16	0.91–1.47	–	–
	3 (high)	746	42.9	–	–	1		–	–
Day of surgery	Monday	225	12.9	1		1		–	–
	Tuesday	284	16.3	**2.82**	**1.24–6.40**	**1.77**	**1.19–2.65**	–	–
	Wednesday	313	18.0	2.00	0.88–4.57	1.30	0.87–1.94	–	–
	Thursday	334	19.2	1.76	0.77–4.00	1.39	0.94–2.06	–	–
	Friday	305	17.5	1.91	0.83–4.39	1.24	0.83–1.85	–	–
	Weekend	277	15.9	1.38	0.58–3.32	1.15	0.76–1.74	–	–
				**C-stat**	**Range**	**C-stat**	**Range**	**C-stat**	**Range**
				0.835	0.833–0.840	0.657	0.651–0.665	0.732	0.727–0.742

^a^ adjusted for: age, Charlson comorbidity index, ASA score and urgency.

^b^ adjusted for: age, Charlson comorbidity index, ASA score and urgency.

^c^ adjusted for: age, Charlson comorbidity index, ASA score and location primary tumour.

^d^ other surgeon specialization: vascular, trauma, or lung surgeons. Other: fellow, surgical resident or unknown.

Bold value: a p ≤ 0.05

For severe complications, similar independent risk factors were found (Table 2). Increased risk on severe complications was found for patients not operated by a GI or oncology specialized surgeon (OR 1.61, 95% CI 1.08–2.39) and for patients operated on Tuesday (OR 1.77, 95% CI 1.19–2.65). Also, duration of stay in the operating room was significantly associated with increased risk on severe complication (OR 1.03, 95% CI 1.01–1.06). However, severe complication risk was lower for patients operated in hospitals with the lowest ICU level (OR 0.75, 95% CI 0.42–1.00).

Higher FTR risk was found for patients who did not stay on a specialized ward postoperatively (OR 2.39, 95% CI 1.52–3.75).

### Sensitivity analysis

The complete case analysis showed similar results with estimates going in the same direction but with wider confidence intervals reflecting less power due to fewer included patients (data not shown). The only differences were that in addition higher blood loss was significantly associated with mortality (OR 1.00, 95% CI 1.00–1.00), while surgery on Tuesday was no longer significantly associated (OR 6.55, 95% CI 0.41–104.79), and that surgery on Thursday was now also significantly associated with increased severe complication risk (OR 3.64, 95% CI 1.08–12.19). Also, lower severe complication risk for patients operated in hospitals with the lowest ICU level became non-significant (OR 1.04, 95% CI 0.45–2.41)

## Discussion

The current study set out to further investigate whether we could identify more specific structural factors, related to the organization of care, that increase risks on mortality, severe complication and FTR in emergency colon cancer surgery. The results showed non-specialized operating surgeon as well as recovery on a non-specialized postoperative ward for GI oncology, operating room time and day of surgery were independent risks factors for mortality, severe complications and FTR. Patients treated in hospitals with the lowest ICU level on average had a lower severe complication risk.

Some of these results are consistent with those found in other studies. Our findings suggest worse outcomes in mortality and severe complications when not operated by a GI or oncology specialized surgeon. The association between surgeon specialization and outcomes in colorectal cancer surgery has been studied before [19–23], with findings showing better outcomes in favour of specialized surgeons [20–23]. Due to their specialized surgical training, these surgeons have more extensive expertise regarding pathology, surgical techniques, oncological and complication treatment possibilities, which makes them the preferred surgeon for colon cancer surgery. Our current findings also showed operating room time (the total time a patients spends in the operating room from entering until leaving) to be associated with increased severe complication risk. Previous studies showed prolonged surgery to be associated with complications such as surgical site infections [24]. In the current study the actual surgery time (i.e. from start incision to closing) was not associated with complications. This could point to factors possibly prior and shortly after surgery that might be associated with increased severe complication risk. Whether these are factors related to the complexity of the severe condition which are not included in the case-mix or related to organization of care are currently unclear and warrant further investigation.

Postoperative stay on specialized wards, for example in stroke patients, showed that dedicated wards can provide organized and multidisciplinary care resulting in beneficial effects on patient outcomes [25]. For colorectal cancer patients, a specialized ward could likewise improve outcomes as the care is more centred and specific complications can be recognized earlier, particularly if these do not occur frequently or are specific for this type of surgery, but this has not been shown previously. It is important to note that hospitals without such a specialized ward will not automatically have worse outcomes as the majority of those hospitals also have specialized surgeons and nurses on the wards, but potentially not to the same extent (e.g. 24/7) as on a specialized ward. General hospitals could be too small to establish separate specialized wards. In addition, we registered the ward on which the patient stayed the majority of the time even though a patient may have stayed on a different (specialized) ward for part of their stay. As a result, the total number of patients staying on a specialized ward, irrespective of transfers between wards, might have been higher.

The results regarding ICU level are less straightforward. ICU-level classification in the Netherlands is based on the available capacity, staffing and management [8]. ICU Level 1 provides basic IC care, whereas Level 3 ICU is better suited to deliver more complex care for severely ill patients. The lowest ICU level has previously been found to be associated with a higher risk on FTR [8] in contrast to the current study where we found no significant association between ICU level and FTR. Instead, we found a significantly lower severe complication risk among patients treated in ICU Level 1 hospitals. One of the explanations is that this is a selection of less complex patients that was not captured by our case-mix and other organizational variables. If these patients were for instance in a better health status when admitted for emergency colon cancer surgery, this could result in lower severe complication risks.

In contrast to previous results including all Dutch hospitals [12], we did not find worse outcomes after weekend surgery in the current study. This may be partly explained by the fact that our study population seemed healthier than patients from non-participating hospitals, reflected in lower ASA score and less advanced tumour stage. However, the fact that our study population showed higher severe complication rates compared to the non-participating hospitals does not point in that direction. Moreover, our results showed worse outcomes for patients operated earlier in the week after adjustment for the specific organizational factors not included in the previous study. For these patients, the weekend can still be a critical period as anastomotic leakage, a common and dreadful complication, is frequently discovered on postoperative days 5–8 [26]. This could mean that the higher risk on mortality and FTR after surgery on Tuesday reflects complications such as anastomotic leakage occurring in the weekend. However, recognition of complications and surgical reintervention during weekends was not independently associated with a higher risk on mortality or FTR. This is in contrast with previous research showing that weekends increased the risk for delay in surgical reintervention for anastomotic leakage [27]. It thus remains unclear how to interpret the worse outcomes seen after surgery on Tuesday in the present study. Also, the wide confidence intervals seen in the mortality risk after surgery on Tuesday should be taken into consideration when interpreting the results, which could mean only a slight increase in risk.

The findings that a non-specialized surgeon and non-specialized postoperative ward for GI oncology are associated with worse outcomes might suggest the establishment a dedicated team of healthcare professionals for patients with colon cancer. But this finding should be interpreted in the context that patients from the current study population were operated in an emergency setting, which might be the biggest risk factor of all. Therefore, preventing patients being operated in an emergency setting is likely far more important. One way to achieve this is the early detection of colon cancer by screening programmes, as implemented in 2014 in the Netherlands [28]. This could help diagnosing colorectal cancer sooner and decrease the future number of emergent surgeries for some patient groups. However, the screening programme only includes people aged between 55 and 75 years [28], which stresses the importance to still carefully monitor patients not included in the screening.

The strength of this study lies in collecting detailed data about the organization and processes of care and their association with patient outcomes, which could help to reduce risks and improve the quality of care. Unfortunately, this study also has its limitations. The main limitation is the completeness of (electronic) medical records with potentially some information not documented because it was not recognized as relevant or forgotten. The electronic patient record system varied across participating hospitals, with some hospitals only having paper records, particularly in the early years, and some hospitals being in the process of switching to electronic records. This may have affected the completeness of data and the extent of missing data for some variables. However, with the availability of techniques such as multiple imputations, missing data nowadays is less of a problem in terms of resulting in bias, and the complete case analysis gave similar results. Another limitation may be that only one individual researcher reviewed the medical records, which could result in the possibility of errors in the reviewing process being overlooked and therefore maintained across the whole data collection. On the other hand, having one researcher also means that the data collection is consistently executed in the same way. A final limitation is the fact that this study was not able to include all the hospitals in the Netherlands. Only a selection of hospitals agreed to participate, which could influence the generalizability of the results.

## Conclusion

This study found that surgeon and ward specialization, duration of surgery and day of surgery were independently associated with increased mortality, severe complication and FTR in patients undergoing emergency colon cancer surgery. This might give inputs on how to organize colon cancer care to improve patient outcomes and quality of care. Still, being operated in an emergency setting in itself increases the risk for worse outcomes, making it a priority to prevent surgeries becoming emergent in the first place.

## Data Availability

The data underlying this article are in part provided by the DCRA with permission from participating hospitals for the authors only. Aggregated data are available upon reasonable request to the corresponding author with permission from DCRA and participating hospitals.

## References

[1] Bakker IS , Snijders HS, Grossmann I et al. High mortality rates after nonelective colon cancer resection: results of a national audit. *Colorectal Dis* 2016;18:612–21. 26749028 10.1111/codi.13262

[2] Baer C , Menon R, Bastawrous S et al. Emergency presentations of colorectal cancer. *Surg Clin North Am* 2017;97:529–45. 28501245 10.1016/j.suc.2017.01.004

[3] Kirchhoff P , Clavien PA, Hahnloser D. Complications in colorectal surgery: risk factors and preventive strategies. *Patient Saf Surg* 2010;4:5. 10.1186/1754-9493-4-5 PMC2852382 20338045

[4] Lee CHA , Kong JCH, Heriot AG et al. Short-term outcome of emergency colorectal cancer surgery: results from Bi-National Colorectal Cancer Audit. *Int J Colorectal Dis* 2019;34:63–9. 30269226 10.1007/s00384-018-3169-5

[5] Degett TH , Dalton SO, Christensen J et al. Mortality after emergency treatment of colorectal cancer and associated risk factors-a nationwide cohort study. *Int J Colorectal Dis* 2019;34:85–95. 30327873 10.1007/s00384-018-3172-x

[6] Donabedian A . The quality of care. How can it be assessed? *JAMA* 1988;260:1743–8. 3045356 10.1001/jama.260.12.1743

[7] Almoudaris AM , Clark S, Vincent C et al. Establishing quality in colorectal surgery. *Colorectal Dis* 2011;13:961–73. 20594202 10.1111/j.1463-1318.2010.02355.x

[8] Henneman D , Van Leersum NJ, Ten Berge M et al. Failure-to-rescue after colorectal cancer surgery and the association with three structural hospital factors. *Ann Surg Oncol* 2013;20:3370–6. 23732859 10.1245/s10434-013-3037-z

[9] Sheetz KH , Dimick JB, Ghaferi AA. Impact of hospital characteristics on failure to rescue following major surgery. *Ann Surg* 2016;263:692–7. 26501706 10.1097/SLA.0000000000001414 PMC4777662

[10] Bray BD , Steventon A. What have we learnt after 15 years of research into the ‘weekend effect’? *BMJ Qual Saf* 2017;26:607–10. 10.1136/bmjqs-2016-005793 27903756

[11] Kothari AN , Zapf MA, Blackwell RH et al. Components of hospital perioperative infrastructure can overcome the weekend effect in urgent general surgery procedures. *Ann Surg* 2015;262:683–91. 26366549 10.1097/SLA.0000000000001436 PMC5169423

[12] Huijts DD , Van Groningen JT, Guicherit OR et al. Weekend effect in emergency colon and rectal cancer surgery: a prospective study using data from the Dutch ColoRectal Audit. *J Natl Compr Canc Netw* 2018;16:735–41. 29891525 10.6004/jnccn.2018.7016

[13] Van Leersum NJ , Snijders HS, Henneman D et al. The Dutch surgical colorectal audit. *Eur J Surg Oncol* 2013;39:1063–70. 23871573 10.1016/j.ejso.2013.05.008

[14] Kolfschoten NE , Marang Van De Mheen PJ, Gooiker GA et al. Variation in case-mix between hospitals treating colorectal cancer patients in the Netherlands. *Eur J Surg Oncol* 2011;37:956–63. 21944049 10.1016/j.ejso.2011.08.137

[15] Van Groningen JT , Ceyisakar IE, Gietelink L et al. Identifying best performing hospitals in colorectal cancer care; is it possible? *Eur J Surg Oncol* 2020;46:1144–50. 32178963 10.1016/j.ejso.2020.02.024

[16] Henneman D , Snijders HS, Fiocco M et al. Hospital variation in failure to rescue after colorectal cancer surgery: results of the Dutch Surgical Colorectal Audit. *Ann Surg Oncol* 2013;20:2117–23. 23417434 10.1245/s10434-013-2896-7

[17] Henneman D , Ten Berge MG, Snijders HS et al. Safety of elective colorectal cancer surgery: non-surgical complications and colectomies are targets for quality improvement. *J Surg Oncol* 2014;109: 567–73. 24338627 10.1002/jso.23532

[18] Huebner M , Vach W, Le Cessie S. A systematic approach to initial data analysis is good research practice. *J Thorac Cardiovasc Surg* 2016;151:25–7. 26602896 10.1016/j.jtcvs.2015.09.085

[19] Borowski DW , Kelly SB, Bradburn DM et al. Impact of surgeon volume and specialization on short-term outcomes in colorectal cancer surgery. *Br J Surg* 2007;94:880–9. 17410637 10.1002/bjs.5721

[20] Barbas AS , Turley RS, Mantyh CR et al. Effect of surgeon specialization on long-term survival following colon cancer resection at an NCI-designated cancer center. *J Surg Oncol* 2012;106:219–23. 22105839 10.1002/jso.22154

[21] Oliphant R , Nicholson GA, Horgan PG et al. Contribution of surgical specialization to improved colorectal cancer survival. *Br J Surg* 2013;100:1388–95. 23939852 10.1002/bjs.9227

[22] Smith JA , King PM, Lane RH et al. Evidence of the effect of ‘specialization’ on the management, surgical outcome and survival from colorectal cancer in Wessex. *Br J Surg* 2003;90:583–92. 12734867 10.1002/bjs.4085

[23] Bergvall M , Skullman S, Kodeda K et al. Better survival for patients with colon cancer operated on by specialized colorectal surgeons - a nationwide population-based study in Sweden 2007–2010. *Colorectal Dis* 2019;21:1379–86. 31293019 10.1111/codi.14760 PMC6916325

[24] Cheng H , Chen BP, Soleas IM et al. Prolonged operative duration increases risk of surgical site infections: a systematic review. *Surg Infect (Larchmt)* 2017;18:722–35. 28832271 10.1089/sur.2017.089 PMC5685201

[25] Stroke Unit Trialists’ Collaboration . Organised inpatient (stroke unit) care for stroke. *Cochrane Database Syst Rev* 2013;9:CD000197. 10.1002/14651858.CD000197.pub3 PMC6474318 24026639

[26] Daams F , Luyer M, Lange JF. Colorectal anastomotic leakage: aspects of prevention, detection and treatment. *World J Gastroenterol* 2013;19:2293–7. 23613621 10.3748/wjg.v19.i15.2293 PMC3631979

[27] Doeksen A , Tanis PJ, Vrouenraets BC et al. Factors determining delay in relaparotomy for anastomotic leakage after colorectal resection. *World J Gastroenterol* 2007;13:3721–5. 17659732 10.3748/wjg.v13.i27.3721 PMC4250644

[28] National Institute for Public Health and the Environment (RIVM) . *Bowel Cancer Screening Programme*. https://www.rivm.nl/en/Topics/B/Bowel_cancer_screening_programme (30 October 2020, date last accessed).

